# Bronchopulmonary dysplasia in preterm neonates: Th2-Eosinophilic inflammation and asthma-like features

**DOI:** 10.1038/s41390-025-04144-4

**Published:** 2025-07-10

**Authors:** Alvaro Moreira, Manissa Coleman, Khyzer Aziz, Vanessa Triviño, Lois Randolph, Noah C. Bierwirth, Charles T. Valadie, Shreyas Arya, Justin A. Meunier, Bryan McOmber, Caitlyn Winter, Grace C. Lee, Isabelle Decker, Alisha M. Smith, Matthew Petershack, Cynthia L. Blanco, Przemko Kwinta, Sunil K. Ahuja

**Affiliations:** 1 Department of Pediatrics, Neonatology Regenerative and Precision Medicine Laboratory, University of Texas Health Science Center at San Antonio, San Antonio, Texas; Veterans Administration Center for Personalized Medicine, South Texas Veterans Health Care System, San Antonio, TX USA; 2 Department of Pediatrics, Johns Hopkins University, Baltimore, MD USA; 3 Department of Pediatrics, University of Texas Health Science Center at San Antonio, San Antonio, TX USA; 4 Department of Pediatrics, Dayton Children’s Hospital, Dayton, OH USA; 5 Veterans Administration Research Center for AIDS and HIV-1 Infection and Center for Personalized Medicine, South Texas Veterans Health Care System, San Antonio, TX USA; 6 Department of Medicine, University of Texas Health Science Center at San Antonio, San Antonio, TX USA; 7 Pharmacotherapy Education and Research Center, School of Medicine, University of Texas Health Science Center at San Antonio, San Antonio, TX USA; 8 College of Pharmacy, University of Texas at Austin, Austin, TX USA; 9 Veterans Administration Center for Personalized Medicine, South Texas Veterans Health Care System, San Antonio, TX USA; 10 The Foundation for Advancing Veterans’ Health Research, South Texas Veterans Health Care System, San Antonio, TX USA; 11 Department of Microbiology, Immunology & Molecular Genetics, University of Texas Health Science Center at San Antonio, San Antonio, TX USA; 12 Department of Pediatrics, Jagiellonian University Medical College, Krakow, Poland; 13 Department of Biochemistry and Structural Biology, University of Texas Health Science Center at San Antonio, San Antonio, TX USA

## Abstract

**Background:**

Asthma is the most prevalent pediatric lung disease, characterized by T-helper 2 (Th2) cell activation and associated eosinophilic inflammation. Mounting evidence suggests a similar Th2 skewing in premature neonates who develop bronchopulmonary dysplasia (BPD), a chronic lung disease with overlapping features of asthma. Given that a substantial proportion of neonates with BPD later develop asthma, our study aimed to investigate the association between an asthma-related transcriptomic signature and BPD.

**Methods:**

Using a previously established 10-gene asthma transcriptomic signature, we analyzed data from 111 very-low-birth-weight (VLBW) neonates over the first month of life. Meta-analysis across seven independent datasets confirmed the association of the asthma gene signature with BPD during the first week of life.

**Results:**

The transcriptomic signature predicted BPD severity as early as day of life 5 and stratified disease progression. Validation in an extremely preterm baboon model of BPD revealed elevated plasma concentrations of interleukin (IL)-5 and IL-6, along with increased expression of Th2-driven inflammatory cytokines in lung tissue.

**Conclusion:**

Our findings provide evidence of a shared genetic and immunologic framework between asthma and BPD, offering potential biomarkers for early diagnosis and avenues for targeted therapy.

**Impact:**

- The asthma-related transcriptomic signature predicts the severity of bronchopulmonary dysplasia (BPD) as early as day five of life, providing a potential early biomarker
- Elevated Th2-eosinophil inflammatory markers suggest a shared pathophysiology between BPD and asthma.
- This study highlights the potential for early diagnosis and targeted interventions to improve long-term respiratory outcomes in preterm infants.

## Introduction

Bronchopulmonary dysplasia (BPD) is the most common complication in infants born extremely premature.^1,2^ BPD is characterized by chronic lung injury due to prolonged mechanical ventilation and supplemental oxygen exposure. While these interventions are life-saving, they initiate a continuous cycle of injury and repair within the fragile, developing lungs of premature infants.^3^ The enduring consequences of BPD include prolonged initial hospitalization, frequent re-hospitalizations, impaired pulmonary function that persists into adulthood, and an increased risk of developing asthma-like diseases.^4–9^ Given the substantial impact of BPD on society and affected families, it is imperative to explore innovative approaches to understand modifiable mechanisms underlying this debilitating disease.

Interestingly, while BPD and asthma are distinct pulmonary diseases, they share common risk factors, symptoms, and treatment approaches. Both conditions are preceded by lung injuries marked by underlying airway inflammation.^10^ Early-life events, such as infection and prematurity, impact lung development increase the risk of asthma.^11,12^ Premature infants often have diminished lung capacity, increased airway obstruction, and an early decline in respiratory function, which collectively increase the risk of developing asthma-like symptoms.^13–16^ The risk of developing asthma is further heightened when prematurity is combined with BPD. In fact, many premature infants with BPD are often treated with asthma medications (e.g., albuterol, inhaled steroids) during their initial birth hospitalization. These shared phenotypic characteristics prompt the exploration of the extent to which BPD and asthma resemble each other. For simplicity, in this paper, we refer to asthma-like diseases and symptoms as “asthma.”

Molecularly, both BPD and asthma are characterized by T-helper 2 (Th2) cell eosinophilic inflammation (Table 1), suggesting a potential genetic overlap. Studies show that premature neonates with elevated eosinophil counts in blood and tracheal samples are more likely to develop BPD. Similarly, approximately half of asthmatics receive treatments targeting Th2 eosinophilic inflammation.^17^ Based on these similarities, we hypothesized a shared genetic overlap between BPD and asthma, which we tested using both preclinical and clinical data. Our primary aim was to evaluate the correlation of an asthma-related transcriptomic signature in neonates diagnosed with BPD. Secondary objectives included examining an early Th2-eosinophil signature in neonates by assessing eosinophil counts at birth. In addition, we validated our findings using a baboon model of extreme prematurity.

**Table 1:** Eosinophilic engagement in BPD.

Author	*n*	Source	Outcome
Yang et al.	261	Blood	Severity of eosinophilia correlated with BPD (OR 7.2)
Yen et al.	107	Blood	Eosinophil counts in the first week of life were higher in BPD
Brostrom et al.	43	Blood	↑Eosinophil counts and eosinophil cationic protein (ECP) in BPD
Raghavender et al.	27	Trachea	ECP was higher in neonates with BPD
Yamamoto et al.	17	Blood	↑Eosinophil counts and number of eosinophil nuclei in BPD

Key articles demonstrating the role of eosinophilic engagement in bronchopulmonary dysplasia.

## Materials and methods

### Asthma gene signature

Our asthma gene signature draws from two primary sources. First, the signature was derived from nasal cell transcriptomics from prospective human aeroallergen challenge studies conducted on individuals with asthma.^18^ Peripheral blood immunologic biomarkers served as the second line of reference to the asthma gene signature.^19^ It consists of ten genes associated with Th2-driven eosinophilic inflammation: *UGT2B28, SYNJ1, SORD, PTGDR2, LGALS7, FBN1, TIPARP, KCNJ8, TSPAN11*, and *ADORA*.^18,19^ This genomic signature provides insights into the diagnosis of asthma, its severity, and the likelihood of asthma exacerbations. Specifically, these genes describe the crosstalk between nasal airway inflammation and respiratory epithelial integrity.

To examine the asthma signature in BPD, we first log-transformed the transcriptomic data and normalized gene expression using quantiles. We then identified differentially expressed genes using the DESeq2 package in R. For the analysis of the ten Th2 eosinophilic genes, we calculated z-scores by subtracting the mean expression of each gene across all samples from the individual gene expression, and divided by the standard deviation of all samples. This approach highlights each gene’s deviation from the mean per patient. These deviations were averaged to create an overall score for each subject. For further details, refer to our previous publications.^18–20^

### Transcriptomic data from very low birth weight neonates with BPD (Krakow, Poland)

Between September 1, 2008, and November 30, 2010, a human cohort study was conducted in Krakow, Poland. This study involved collecting peripheral blood samples from a total of 111 infants. In this study, BPD was defined as the requirement for positive pressure ventilation or oxygen at 36 weeks of age.^21^ The inclusion criteria for participants consisted of infants born with a gestational age of less than 32 weeks, birthweight equal to or less than 1500 g (VLBW), and were receiving respiratory support at time of study enrollment.

**Fig. 1:**
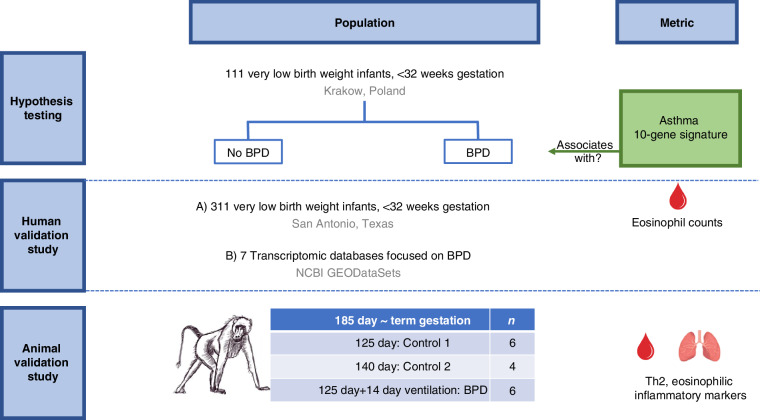
Study design for evaluating the asthma 10-gene signature in BPD. The study included three components: (1) Hypothesis Testing in 111 very low birth weight infants ( < 32 weeks gestation) from Krakow, Poland, to assess the association of the asthma 10-gene signature with BPD; (2) Human Validation using 311 infants from San Antonio, Texas, and seven BPD-focused transcriptomic datasets to confirm findings and evaluate eosinophil counts; and (3) Animal Validation in a baboon model with preterm controls and a BPD group (125-day gestation + 14 days ventilation) to analyze Th2 and eosinophilic inflammation.

Peripheral blood samples were collected from all participants on days 5, 14, and 28. RNA was extracted from the peripheral blood mononuclear cells to obtain microarray gene expression data. The study tracked 75 of these neonates until they reached four years old to report any wheezing incidents post-discharge from the neonatal intensive care unit (NICU). Figure 1 provides an overview of the study design.

### Meta-analysis of transcriptomic data from very low birth weight neonates with BPD

To further explore the relevance of the asthma gene signature in the context of BPD, we conducted a meta-analysis of seven publicly available microarray or RNA sequencing datasets. These datasets focused on BPD and included transcriptomic data from neonate. To harmonize the data, we log-transformed the gene expression values, filtered for common genes across datasets, and performed quantile normalization. Batch effects were corrected using the normalizeBetweenArrays() and removeBatchEffect() functions from the limma R package. Cell type enrichment analysis was then performed using the xCell R package, which incorporates gene signatures from 64 immune and stromal cell types.

The analysis also aimed to identify immune cell populations enriched in BPD versus non-BPD samples. Data were derived from samples collected during the first week of life, as most datasets only included a single time point. A Wilcoxon rank-sum test was used to compare cell abundance between groups, preceded by a test of normality to validate the distribution of the data. Detailed descriptions of the datasets and methodologies can be found in the Supplement.

### Human validation cohort (Texas, USA)

To investigate findings of Th2 eosinophilic inflammation in BPD, we included 311 VLBW neonates admitted to the NICU at University Hospital in San Antonio between January 2014 and December 2018. At birth, we collected peripheral blood absolute eosinophil counts (cells/µL) of neonates with a gestational age of less than 32 weeks. By replicating the specific birth weight and gestational age range observed in Poland’s study, we aimed to ensure the relevance and generalizability of our findings across different demographics. Permissions to include this subset of infants was provided by the local institutional review board via protocol number HSC20200175H.

### Non-human primate model mimicking BPD

To study the microarray findings, we analyzed 16 samples from banked tissues of our non-human primate baboon model. Pregnant baboons (Papio sp.) were procured from the Southwest Biomedical Research Center and subsequently transported to the baboon neonatal intensive care unit. Baboons were delivered via cesarean section at different timepoints: (i) 125-day gestation ( ~ 25–27 weeks in humans, *n* = 6, Control 1), (ii) 140-day gestation ( ~ 28–31 weeks in humans, *n* = 4, Control 2), and (iii) at 125-day gestation followed by 14 days of mechanical ventilation and exposure to hyperoxia to induce a BPD-like phenotype (*n* = 6). Serum analytes from the animal BPD cohort were collected at birth, and lung RNA samples were obtained at time of necropsy (day 14), whereas control animals were euthanized shortly after birth.

#### Lung tissue

Lung RNA analysis was conducted using reverse transcription-polymerase chain reaction (RT-PCR) to measure the relative mRNA expression of genes associated with the Th2-eosinophil inflammatory pathway in snap-frozen lung tissue. Approximately 20 mg of tissue was used for RNA extraction, following the Qiagen RNeasy minikit protocol (Qiagen). Tissue disruption was achieved by bead beating with 0.7 mm zirconia beads (BioSpec Products, Bartlesville) in two 1.5 min homogenization steps, separated by a 1 min rest on wet ice. RNA quantity was determined using a BioTek Epoch spectrophotometer with a Take3 plate (BioTek Instruments, Winooski).

For cDNA synthesis, 2 ng of RNA were used as a starting quantity with the Applied Biosystems High Capacity cDNA transcription kit (Thermo Fisher Scientific, Foster City) following the manufacturer’s instructions. Relative gene expression of interleukin 5 (*IL-5*), interleukin 13 (*IL-13*), serpin family B member 2 (*SERPIN B2*), and chloride channel accessory 1 (*CLCA1*) were measured using KiCqStart SYBR Green primer/probe sets and master mix (MilliporeSigma, Burlington) on the BioRad CFX384 Touch Real-Time PCR Detection System (BioRad Laboratories, Hercules). Gene expression quantification utilized the relative standard curve method, with gene quantities normalized to importin 8 (IPO8).

#### Blood tissue

Plasma concentrations of Th2 inflammatory cytokines were determined using the MILLIPLEX-MAP Non-Human Primate Cytokine Magnetic Bead Panel - Immunology Multiplex Assay (MilliporeSigma, Burlington, CAT: PRCYTOMAG-40k) following the manufacturer’s protocol. Quantification was performed with the Luminex FlexMAP 3D instrument (Luminex Corporation, Austin) and xPONENT software (v4.2.1441.0, Luminex Corporation).

### Statistical analysis

#### Hypothesis testing

We compared the z-score expression levels of the asthma transcriptomic signature between the non-BPD and BPD groups. To assess transcriptomic differences, we employed generalized linear models (GLMs) with generalized estimating equations (GEEs), treating time as a categorical variable. The data were visualized using line plots, displaying mean values and standard errors of the mean (SEM), with *p*-values indicating differences in gene expression over time. We also assessed variations in BPD severity over time, analyzing the progression of transcriptomic differences in relation to clinical outcomes.

To evaluate the predictive capacity of the asthma gene signature, we randomly divided the data into training and testing datasets (75:25 split). Subsequently, we constructed a logistic regression model utilizing the asthma gene z-score at day 5 as the independent variable and BPD as the dependent variable. We assessed the model’s performance in the testing dataset by calculating sensitivity, specificity, and the area under the receiver operating characteristic curve (AUC).

#### Human validation study

Categorical variables were presented as frequencies with percentages. Continuous data were described as medians with interquartile ranges (IQR) or means with standard deviations. To compare medians or means, we employed the Wilcoxon rank-sum test or Welch’s test, while categorical data were analyzed using the χ^2^ test or Fisher’s exact test. A *p*-value less than 0.05 was considered indicative of statistical significance.

#### Animal validation study

A one-way ANOVA was used to analyze continuous variables in the three animal groups, followed by post hoc analysis using Welch’s test. The Wilcoxon rank-sum test was used to compare data described as median with IQs. To control for false discovery rates, we applied Bonferroni correction to adjust *p*-values. Significance was determined at a threshold of *p* < 0.05.

## Results

### Asthma gene signature associates with BPD and long term wheezing

Among the study participants, 29 (26.1%) met the criteria for BPD. Neonates who developed BPD had a lower median gestational age and birthweights, [25.5 weeks (IQR, 24.75, 27.0) vs. 28.0 weeks (IQR, 26.0, 30.0), *p* < 0.01 weeks] and [1120 grams (IQR, 870, 1,300) vs. 705 grams (IQR, 605, 899), *p* < 0.001].^22^

**Fig. 2:**
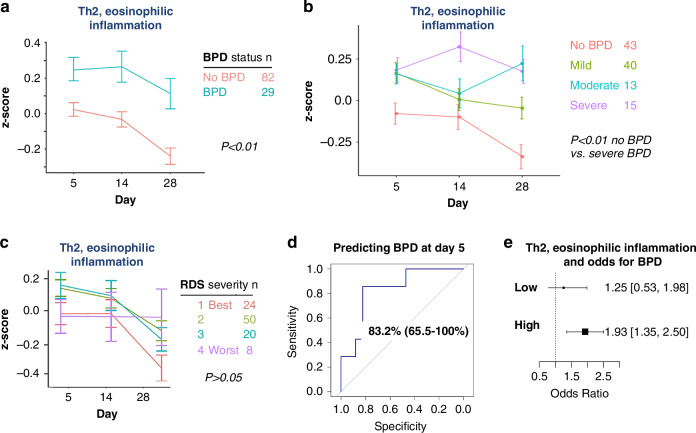
Asthma gene signature and its association with BPD development. **a** Patients diagnosed with BPD consistently exhibit higher z-scores for Th2 inflammation, as determined by our 10-gene asthma signature, over the first month of life, compared to those without BPD (*P* < 0.01). **b** Th2 inflammation z-scores are stratified by BPD severity (NICHD 2001 definition), showing a significant difference between patients with no BPD and those with severe BPD (*P* < 0.01, no BPD vs. severe BPD). **c** Patients with more severe initial lung disease, as evaluated by the severity of RDS, do not necessarily exhibit increased expression of the 10-gene asthma signature (*P* > 0.05). **d** Receiver Operating Characteristic (ROC) curve demonstrating an area under the curve (AUC) of 83.2% (65.5%–100%) for predicting BPD at day 5 using the asthma gene signature. **E** Odds ratio for developing BPD based on low or high expression of the asthma gene signature. High expression is associated with an increased risk (OR 1.93, 95% CI [1.35, 2.50]), while low expression shows no significant association (OR 1.25, 95% CI [0.53, 1.98]).

Figure 2a shows the mean z-score for the asthma gene signature across the first month of life. Neonates with BPD exhibited significantly higher expression of Th2 eosinophilic inflammatory genes compared to those without BPD. Stratification by BPD severity revealed a progressive increase in gene expression among neonates with severe BPD, with marked differences emerging by Day 14 and becoming more pronounced by Day 28 (Fig. 2b).

To determine the specificity of the asthma gene signature for BPD, its association with respiratory distress syndrome (RDS) severity, as assessed by radiologic scoring, was examined (Fig. 2c). The asthma gene signature demonstrated strong predictive ability for BPD, with an area under the receiver operating characteristic curve (AUC) of 83.2% (95% CI, 65.5–100%) by day five, along with a sensitivity of 85.7% and specificity of 82.4% (Fig. 2d). When stratified by gene expression levels on day five (high vs. low, based on the median), neonates with high expression had an odds ratio of 1.93 (95% CI, 1.35–2.50) for developing BPD (Fig. 2e).

Seventy-five neonates (67.6%) were monitored for the first four years of life. Among them, 41 (54.7%) experienced at least one episode of wheezing. The 14-days asthma gene signature was associated with the occurrence of wheezing episodes; odds ratio 3.5, with a 95% confidence interval ranging from 1.1 to 12.4 (*p* = 0.03). Need to check with the new breakdown

### Meta-analysis of asthma gene signature across 7 datasets

To validate the relevance of the asthma gene signature in BPD, we conducted a meta-analysis of transcriptomic data across seven independent datasets (Supplemental Table S1). The analysis focused on the first week of life, encompassing 10 genes associated with Th2-driven eosinophilic inflammation. The z-score distribution of the asthma gene signature was significantly elevated in neonates with BPD compared to those without BPD (*p* = 3.484e-07, Supplemental Fig. S1). This finding confirms a robust association between the asthma gene signature and BPD across multiple datasets, supporting its generalizability to diverse cohorts.

### Neutrophil and eosinophil enrichment in the first week of life

Immune cell enrichment analysis identified significant differences in neutrophil and eosinophil abundance between neonates with and without BPD during the first week of life. Neutrophil abundance was markedly higher in neonates with BPD compared to non-BPD neonates (*p* < 0.01, Supplemental Fig. S2, left), suggesting a strong neutrophilic inflammatory response in the early stages of BPD development. Similarly, eosinophil abundance was significantly elevated in neonates with BPD (*p* < 0.01, Supplemental Fig. S2, right), highlighting the potential contribution of Th2 eosinophilic inflammation to disease pathology. These findings highlight the role of innate and adaptive immune responses in the early inflammatory environment of BPD.

### Th1, Th2, and CD4/CD8 immune responses in the first week of life

To further characterize immune responses in BPD, we analyzed Th1, Th2, CD4, and CD8 T-cell abundances across the meta-analysis datasets during the first week of life. Th1 cell abundance was reduced in neonates with BPD compared to those without (Supplemental Figure S3, top left panel). In contrast, Th2 cell abundance did not differ significantly between groups (*p* = 0.31, Supplemental Figure S3, top right panel). Similarly, CD4 T-cell abundance showed no significant difference between BPD and non-BPD neonates (*p* = 0.73, Fig. 1c, bottom left panel). However, CD8 T-cell abundance was markedly increased in neonates with BPD (*p* < 0.01, Fig. 1c, bottom right panel).

### Elevated baseline eosinophil counts in BPD neonate

**Fig. 3:**
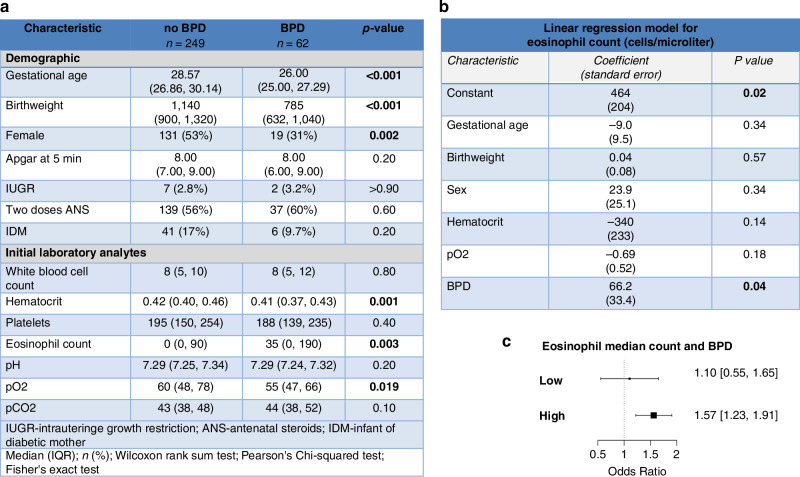
Human validation cohort. **a** Characteristic table in human validation cohort. **b** Linear regression model for eosinophil count with independent variables selected by univariate analysis. Only BPD remains a statistically significant predictor after regression analysis. **c** Odds ratio for developing BPD amongst VLBW neonates based off of low or high peripheral eosinophil count.

An external cohort of neonates from University Health System in San Antonio, Texas, confirmed the association between eosinophilic inflammation and BPD. Infants who developed BPD were of lower gestational age (26.0 vs. 28.57 weeks, *p* < 0.01) and had a lower birthweight (785 grams vs. 1140 grams, *p* < 0.01). They also had higher peripheral blood eosinophil counts at birth [0 (IQR, 0, 90) cells/µL compared to 35 (IQR, 0, 190) cells/µL, *p* = 0.003; see Fig. 3a].

In a multivariable linear regression model, early demographic variables and laboratory parameters were assessed for their association with eosinophil counts. BPD diagnosis was significantly associated with elevated eosinophil counts, while demographic variables such as gestational age and partial oxygen pressures did not achieve statistical significance (Fig. 3b). Neonates with eosinophil counts above the median had an increased risk of developing BPD, with an odds ratio of 1.57 (95% CI, 1.23–1.91; Fig. 3c).

### Validation in a premature baboon model of BPD

**Fig. 4:**
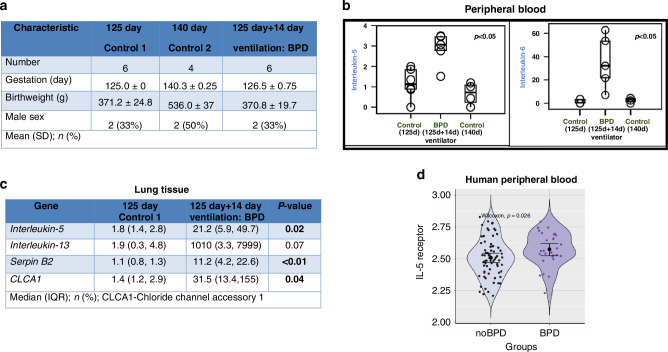
Animal validation cohort. **a** Characteristics of our animal validation cohort. **b** Serum concentrations of intraleukin-5 and intraleukin-6 are significantly higher amongst the chronically intubated BPD model compared to both preterm and term controls. **c** mRNA expression of Th2 inflammatory markers IL-5, Serpin B2, and CLCA1 are significantly elevated in the chronically intubated BPD model compared to preterm controls. IL-13 expression is not different. **d** Violin plot showing gene expression of IL-5 receptor antagonist in Polish cohorts based on BPD status. Analysis per Wilcoxon rank sum test.

To corroborate these findings, we examined the association between Th2-eosinophil inflammatory markers and BPD in a premature baboon model. Six preterm baboons subjected to chronic ventilation and intubation for 14 days demonstrated significant increases in serum Th2 cytokines, including IL-5 and IL-6, compared to unventilated controls (*p* < 0.05; Fig. 4b). Transcriptomic analysis of lung RNA from the BPD baboon cohort revealed elevated expression of Th2-eosinophil markers, such as *IL-5, Serpin B2*, and *CLCA1* (Fig. 4c). Although IL-13 exhibited a trend toward higher expression, it did not reach statistical significance. These findings were consistent with the Polish neonatal cohort, where IL-5 expression in peripheral blood was significantly elevated in neonates with BPD (Fig. 4d).

## Discussion

In this comprehensive translational study, we established a link between Th2-driven eosinophilic inflammation and the development of BPD. Although BPD and asthma are distinct conditions, they share key immunological and phenotypic similarities. For simplicity, this paper refers to asthma-like diseases in BPD as “asthma.” Our results add to the understanding of these overlapping conditions. Although asthma is characterized by chronic pulmonary inflammation, heightened airway responsiveness, and a Th2-driven cytokine profile, the evidence for similar processes in BPD remains less established. Many BPD neonates are managed with medications traditionally used in asthma (e.g., albuterol, inhaled corticosteroids), but the supportive data for such treatment remains limited. The shared molecular pathways identified in this study may help explain this overlap and justify further research into targeted interventions that address eosinophilic inflammation in BPD.

### Th2 responses in the first week of life and meta-analysis findings

Despite the strong association between eosinophilic inflammation and BPD, our meta-analysis of seven independent datasets did not show significantly elevated Th2 cell abundance in the first week of life in neonates with BPD. This finding may reflect the early suppression of adaptive immune responses, as evidenced by the significant reduction in Th1 cells observed in BPD neonates. The lack of an early Th2 elevation could also be explained by compensatory immunologic shifts triggered by perinatal stressors such as hyperoxia, infection, or mechanical ventilation.^23^ These stressors may initially suppress adaptive immunity, allowing innate immune responses, like eosinophilic and neutrophilic inflammation, to dominate.

Alternatively, Th2-skewed immune responses may emerge later in the neonatal period as part of an inflammatory cascade that evolves with ongoing pulmonary injury.^24^ These findings are consistent with prior studies indicating that a Th2 bias is present during fetal life and early neonatal development to promote immune tolerance.^25–27^ However, in preterm neonates, environmental insults may exacerbate this Th2 bias, leading to maladaptive pulmonary inflammation and injury. Longitudinal studies are needed to determine the timing and progression of Th2 activation and its contribution to BPD pathogenesis.

### Eosinophil-driven inflammation as a predictor of BPD

A key finding of our study is the significant elevation of eosinophils in the first week of life in neonates with BPD, consistent across multiple datasets and validation cohorts. Our results align with previous findings by Yen et al., who reported higher eosinophil counts in VLBW infants with BPD.^28^ Expanding on this work, we demonstrate that eosinophil counts on the first day of life can serve as a predictive marker for BPD, even after accounting for known risk factors.^29–32^

The early elevation in eosinophils suggests a prominent role for the innate immune system in BPD pathogenesis. While eosinophils are classically associated with Th2 responses, their early rise may occur independently of adaptive immune activation, possibly driven by antenatal inflammation or perinatal stressors.^33^ This finding highlights the potential of eosinophil-driven inflammation as a critical target for early interventions aimed at mitigating pulmonary injury in preterm neonates.

### Th1 suppression and CD8 expansion in BPD

Our analysis revealed a significant reduction in Th1 cell abundance in neonates who developed BPD, a seemingly paradoxical finding given the heightened inflammation typically seen in preterm neonates. This suppression may reflect impaired adaptive immune regulation, with preterm neonates exhibiting immature T-cell maturation and cytokine production.^34,35^ Alternatively, the reduction in Th1 cells could represent an adaptive mechanism to limit pro-inflammatory damage in developing lungs.

Interestingly, the suppression of Th1 responses coincides with a significant expansion of CD8 T cells in BPD neonates. Elevated CD8 T-cell levels suggest heightened cytotoxic activity, which may exacerbate tissue damage and amplify pulmonary injury.^36^ Together, these findings highlight a complex immune dysregulation in BPD, characterized by Th1 suppression, CD8 expansion, and eosinophilic inflammation. This interplay of immune responses emphasizes the importance of understanding how compensatory and pathogenic mechanisms contribute to BPD progression.

### Validation in a preterm baboon model

Our findings were validated in a preterm baboon model, which recapitulates key features of BPD, including airway reactivity, pulmonary hypertension, and parenchymal dysfunction. Baboons with a BPD phenotype exhibited elevated Th2 cytokines, such as IL-5 and IL-6, in serum and lung tissues, as well as increased expression of eosinophilic inflammatory markers, including *IL-5, Serpin B2*, and *CLCA1*, in lung transcriptomes. These findings provide strong translational evidence for the role of Th2-driven eosinophilic inflammation in BPD and suggest that life-saving interventions like mechanical ventilation and supplemental oxygen may perpetuate this inflammatory cascade. This model highlights the importance of identifying neonates at high risk for BPD to implement targeted therapeutic interventions.

### Clinical implications and future directions

Our study provides insights into the immunopathology of BPD, emphasizing the role of Th2-driven eosinophilic inflammation as both a biomarker and potential therapeutic target. The absence of early Th2 elevation in our meta-analysis underscores the need for longitudinal studies to track immune dynamics over time. Understanding the temporal relationship between Th2 activation, eosinophil-driven inflammation, and disease progression will be essential for developing early and potentially effective interventions.

Given the overlap between eosinophilic inflammation in BPD and other chronic lung diseases, such as asthma and asthma-COPD overlap syndrome (ACO), targeted therapies may hold promise.^37–39^ For instance, IL-5-targeted treatments, which are effective in eosinophilic asthma, could be repurposed for BPD. Early trials of IL-5Rα inhibitors like benralizumab in preterm animal models could pave the way for personalized approaches to treat eosinophilic inflammation in neonates with chronic lung disease.^38,40^

### Limitations

Despite the strengths of our large cohort and preterm baboon model, our study has several limitations. The a priori 10-gene panel, while designed to assess asthma-related inflammation, may not capture all relevant gene signatures reflective of asthma or wheeze, potentially overlooking other pathways contributing to BPD pathogenesis. Transcriptomic assessments and eosinophil counts were not performed in the baboon model, which would have strengthened the translational relevance of our findings. Additionally, the absence of data on key prenatal risk factors, such as chorioamnionitis and premature rupture of membranes, limits our ability to control for potential confounders. Finally, as a retrospective study, our findings require validation through prospective cohort studies to establish causality and refine biomarkers for BPD prediction.

## Conclusions

In conclusion, our study provides evidence of a significant role for eosinophilic inflammation in the development of BPD, with elevated eosinophil counts evident as early as the first week of life. While Th2-driven inflammation is implicated in BPD pathogenesis, the absence of early Th2 cell elevation in our meta-analysis suggests a complex and dynamic temporal pattern of immune activation. These findings emphasize the need for further research to define the timing and mechanisms of Th2 activation and to evaluate the potential of eosinophilic inflammation as a predictive biomarker and therapeutic target for BPD.

## Supplementary information

Supplementary information

## Data Availability

The transcriptomic data set of ELBW infants with BPD is publicly available and can be accessed at via NCBI Gene Expression Omnibus by the following address https://www.ncbi.nlm.nih.gov/geo/query/acc.cgi?acc=GSE32472. The data for our human validation cohort is not readily available due to concern that these data may compromise the privacy of participants. However, the data may be obtained from corresponding author Dr. Alvaro Moreira upon reasonable request.
