# Supplementary material for: Bronchopulmonary dysplasia in preterm neonates: Th2-Eosinophilic inflammation and asthma-like features
Source: Pediatr Res. 2025 Jul 10;99(3):921–8. doi: 10.1038/s41390-025-04144-4 (PMC13021504; doi:10.1038/s41390-025-04144-4)

SUPPLEMENTAL FILES

Supplemental Table S1: Summary of transcriptomic datasets used for asthma gene signature analysis in the first week of life.

This table lists the seven transcriptomic datasets included in the study, detailing the study authors, publication year, and the number of neonates analyzed during the first week of life. The datasets range from 2007 to 2023, with sample sizes varying from 10 to 97 patients per dataset, providing a comprehensive and diverse cohort for assessing the asthma gene signature in relation to BPD.

Study	Year	# of patients in 1st week of life
GSE8586 (Kohane I et al)	2007	54
GSE32472 (Kwinta P et al)	2011	*97*
GSE188944 (Bell D et al)	2022	23
GSE225881 (Hossain H et al)	2023	61
GSE106910 (Spadafora R et al.	2018	54
GSE125873 (Ryan FJ et al)	2019	10
GSE220135 (Bell D et al)	2023	68

Supplemental Figure S1: Asthma gene signature z-scores in neonates with and without BPD during the first week of life.

This boxplot summarizes the z-scores of the asthma 10-gene signature across all seven datasets during the first week of life, comparing neonates with BPD (blue) to those without BPD (red). Neonates with BPD exhibit significantly higher z-scores, indicating greater expression of Th2 eosinophilic inflammatory genes (p-value = 3.484e−07*). Normality was confirmed using Shapiro's test, and statistical significance was determined using a t-test.

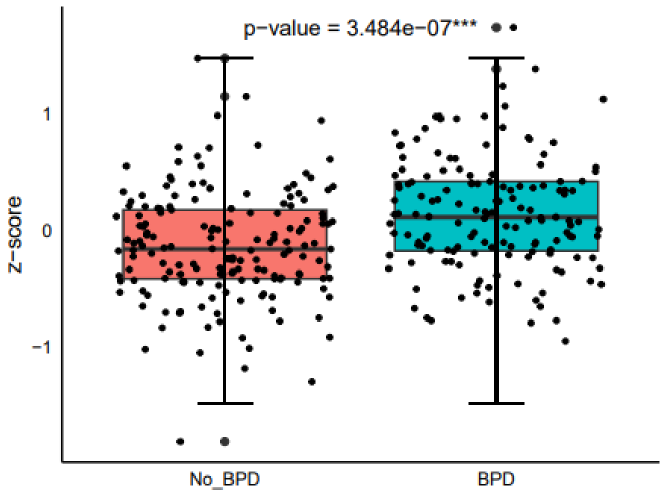

Supplemental Figure S2: Increased neutrophil and eosinophil abundance in neonates with BPD.

Left: Neutrophil cell abundance is significantly higher in neonates with BPD compared to those without BPD (p-value = 0.0007738). Right: Eosinophil cell abundance is also significantly elevated in neonates with BPD (p-value = 0.005062).

These violin plots illustrate the distribution of cell abundances, with higher inflammatory cell levels observed in the BPD group, supporting a role for neutrophilic and eosinophilic inflammation in BPD development.

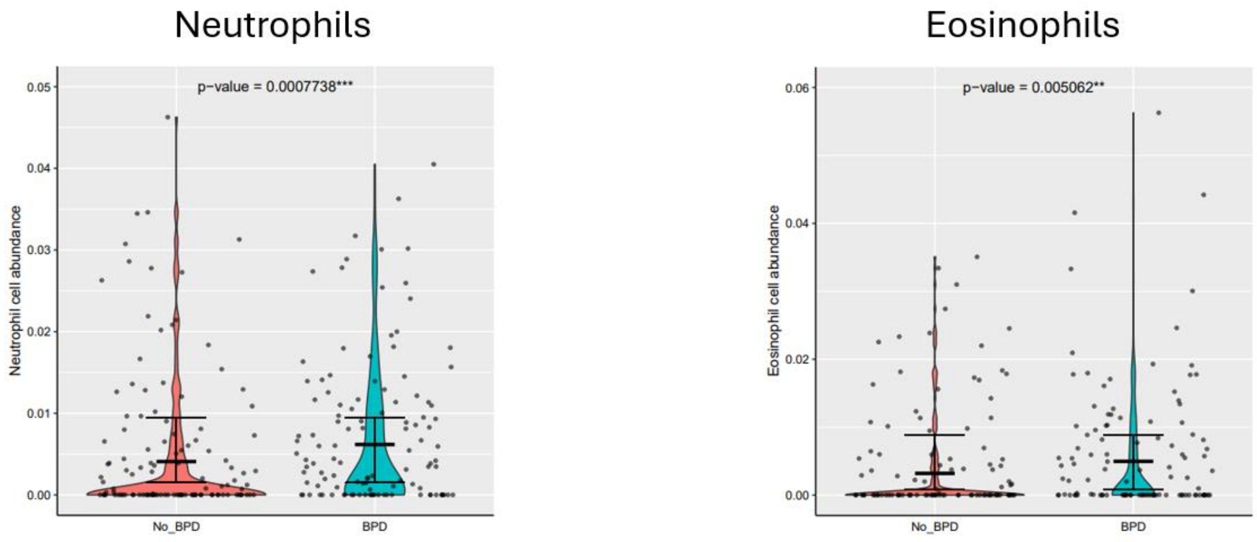

Supplemental Figure S3: Differential T cell subset abundance in neonates with and without BPD.

Top Left: Th1 cell abundance is significantly higher in neonates with BPD compared to those without (p = 0.006177). Top Right: Th2 cell abundance shows no significant difference between neonates with and without BPD (p = 0.3068). Bottom Left: CD4+ T cell abundance is comparable between the two groups (p = 0.73). Bottom Right: CD8+ T cell abundance is significantly elevated in neonates with BPD (p = 0.007585).

These violin plots demonstrate that specific T cell subsets, including Th1 and CD8+ T cells, are enriched in neonates with BPD, highlighting their potential role in disease pathogenesis.

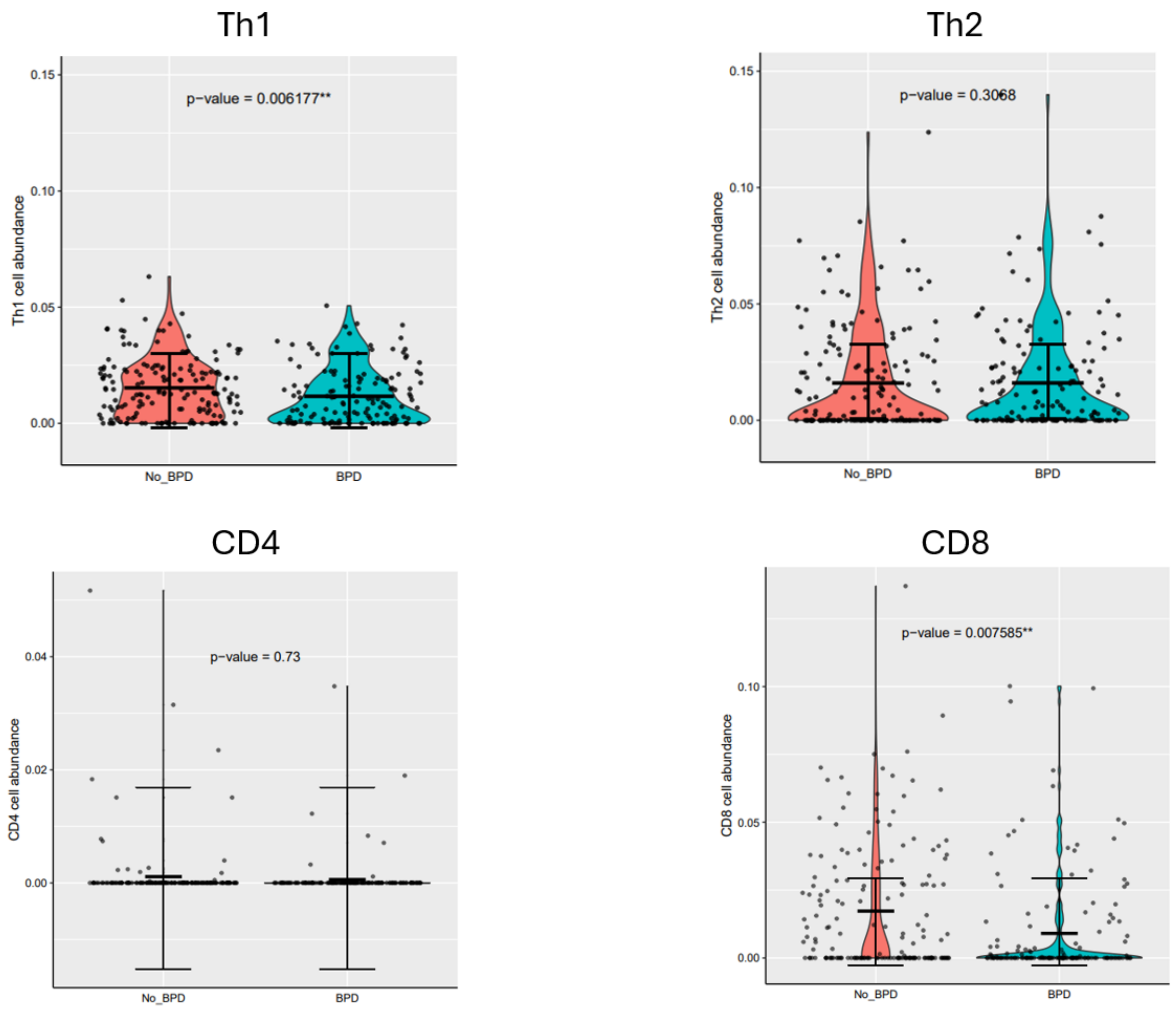

Supplement: Supplementary file 1 — Supplementary information [file 41390_2025_4144_MOESM1_ESM.pdf]
